# Nonlinear Imaging Detection of Organ Fibrosis in Minute Samples for Early Stage Utilizing Dual-Channel Two-Photon and Second-Harmonic Excitation

**DOI:** 10.3390/bios15060357

**Published:** 2025-06-04

**Authors:** Bo-Song Yu, Qing-Di Cheng, Yi-Zhou Liu, Rui Zhang, Da-Wei Li, Ai-Min Wang, Li-Shuang Feng, Xiao Jia

**Affiliations:** 1 School of Instrumentation and Optoelectronic Engineering, Beihang University, Beijing 100191, China; bosongyu@buaa.edu.cn; 2 Zhongshan Institute for Drug Discovery, Chinese Academy of Sciences, Zhongshan 528400, China; chengqingdi0603@zidd.ac.cn; 3 Department of Kidney Transplantation, The Third Affiliated Hospital of Sun Yat-sen University, Guangzhou 510630, China; zhangr97@mail2.sysu.edu.cn; 4 School of Information Science and Engineering, Shandong Provincial Key Laboratory of Laser Technology and Application, Shandong University, Qingdao 266237, China; yizhou.liu@sdu.edu.cn; 5 Key Laboratory of Laser & Infrared System Ministry of Education, Shandong University, Qingdao 266237, China; 6 College of Future Technology, Peking University, Beijing 100871, China; lidawei@pku.edu.cn; 7 School of Electronics, Peking University, Beijing 100871, China; wangaimin@pku.edu.cn; 8 State Key Laboratory of Advanced Optical Communication System and Networks, School of Electronics, Peking University, Beijing 100871, China; 9 Key Laboratory of Precision Opto-Mechatronics Technology (Ministry of Education), Beihang University, Beijing 100191, China; 10 State Key Laboratory of Drug Research, Shanghai Institute of Materia Medica, Chinese Academy of Sciences, Shanghai 201203, China

**Keywords:** nonlinear imaging, two-photon excitation, second harmonic generation, fibrosis

## Abstract

Histopathological staining remains the fibrosis diagnostic gold standard yet suffers from staining artifacts and variability. Nonlinear optical techniques (e.g., spontaneous fluorescence, Second Harmonic Generation) enhance accuracy but struggle with rapid trace-level detection of fibrosis. To address these limitations, a dual-channel nonlinear optical imaging system with excitation wavelengths at 780 nm and 820 nm was developed, enabling simultaneous spontaneous fluorescence and second-harmonic generation imaging through grid localization. This study applies dual-modality nonlinear imaging to achieve label-free, high-resolution visualization of pulmonary and renal fibrosis at the ECM microstructure scale. Through leveraging this system, it is demonstrated that collagen can be rapidly detected via spontaneous fluorescence at 780 nm, whereas the spatial distribution of collagen fibrils is precisely mapped using Second Harmonic Generation at 820 nm. This approach allows for the rapid and sensitive detection of trace fibrosis in a 5-day unilateral ureteral obstruction mouse model. Additionally, we identify that the elastic fibers, which can also be visualized, provide a foundation for staging diagnosis and delivering accurate and quantitative data for pathological studies and analysis. The research findings underscore the potential of this dual-channel nonlinear optical imaging system as a powerful tool for rapid, precise, and noninvasive fibrosis detection and staging.

## 1. Introduction

Being a pathological hallmark of chronic tissue injury, organ fibrosis is driven by the excessive deposition of extracellular matrix (ECM) components, which results in progressive architectural distortion, functional impairment, and eventual organ failure [1,2,3]. Accurate staging and biomarker discovery play a crucial role in guiding therapeutic strategies [4,5,6,7], while conventional diagnostic approaches, including biopsy-based histopathology and immunofluorescence staining for renal and pulmonary fibrosis, remain constrained by technical variability (e.g., stain consistency and subjective interpretation) and limited sensitivity to early fibrotic changes or dynamic ECM remodeling during the process of treatment [4,7,8,9,10,11,12]. Therefore, alternative and more informative imaging modalities could be utilized to facilitate an accurate and rapid assessment of therapeutic interventions in fibrosis.

In response, nonlinear optical imaging modalities, including Second Harmonic Generation (SHG) and Two-Photon Excitation Fluorescence (TPEF), have emerged as transformative tools for label-free and high-resolution visualization of ECM dynamics [13,14,15]. Certain studies have identified that label-free SHG imaging provides greater sensitivity in detecting fibrillar collagen deposition within organ fibrosis compared to conventional histological staging [16,17]. According to James et al., microscopy effectively characterizes altered collagen architecture in 3D idiopathic pulmonary fibrosis spheroid models under cross-linking modulation, while Lee et al. revealed that aged lungs exhibit heightened interstitial collagen density, reduced alveolar expansion, and diminished surfactant secretion through SHG imaging, which collectively underscored SHG’s utility in profiling fibrotic pathologies [18,19]. Moreover, TPEF employs specific light to deeply excite fluorophores in the tissue, which not only minimizes autofluorescence (AF) but also enhances the detection of endogenous or exogenous markers associated with fibrosis [13,16].

Innovative approaches for early detection have been demonstrated by the recent advancements in organ fibrosis research. Notably, the integration of molecular probes with two-photon excitation microscopy has already become a promising strategy, leveraging probe-bound collagen deposition to generate detectable fluorescence signals indicative of incipient fibrotic changes. Fan et al. developed an MAO-B-activated two-photon probe (BiPhAA) for rapid (<10 min) hepatic stellate cell imaging [20], while Zhou designed a PET-based NO probe (PYSNO) with nanomolar sensitivity to track myocardial fibrosis [21], both of which risk disturbing the native collagen architecture. However, despite these technological breakthroughs, the direct utilization of collagen’s AF for early fibrosis detection is still substantially underexplored. In fibrotic tissues, regions of pathological interest are sparsely distributed compared to adjacent normal areas, which leads to attenuated or absent imaging signals and consequent false-negative diagnoses in clinical assessments [22].

In contrast to the well-characterized role of collagen, elastin is understudied despite its emerging diagnostic relevance. Recent advances demonstrate that elastin quantification promotes reproducible assessment of renal fibrosis progression and longitudinal evaluation of antifibrotic therapeutic efficacy [23,24,25]. However, persistent technical limitations are faced by the integration of elastin analysis with nonlinear optical imaging modalities. Firstly, the lower physiological abundance of elastin compared to collagen renders its intrinsic contrast often insufficient for label-free histopathological imaging [24]. Secondly, there is a dearth of studies investigating whether elastin can be used as a biomarker for various stages or types of fibrosis. Although higher-order harmonic generation (SHG/THG) is wavelength-independent in principle, efficient excitation requires specific wavelength ranges. Below 800 nm, the emission spectrum of collagen simultaneously encompasses both SHG peaks and two-photon emission signals [26,27]. While its intensity decreases as the excitation wavelength increases, this two-photon fluorescence can interfere with the accurate detection of collagen. The collagen no longer generates two-photon fluorescence above 820 nm, and excitation >820 nm is required to localize the SHG signal from collagen [28,29]. However, elastin, a vital component for detection, demonstrates an optimal excitation range of 780–800 nm, encountering signal attenuation at longer wavelengths. Therefore, 820 nm balances collagen’s SHG specificity and residual elastin detectability.

However, the implementation of nonlinear imaging critically depends on high-performance lasers, which provide intense and tunable light sources essential for exciting nonlinear optical effects [30]. Due to the advancing tunable ultrafast optical technology, the generation of the required ultrafast imaging light source is enabled. Typically, the nonlinear phase modulation can be employed to realize a nonlinear frequency shift, followed by frequency doubling to provide a tunable ultrafast light source for collagen fiber and elastin imaging [31,32,33,34,35]. By carefully tuning the laser parameters, the corresponding imaging sensitivity of the SHG can be enhanced and, therefore, ensure imaging accuracy [35]. The imaging system ultrafast lasers are used to concurrently map collagen fibrils through 780 nm AF and visualize fibrillar organization via 820 nm SHG microscopy at submicron resolution. This integrated approach enables precise collagen localization (early stage) while overcoming SHG signal attenuation, which hence ensures robust detection across heterogeneous tissue structures.

These findings demonstrate the clinical utility of dual-wavelength SHG imaging in fibrosis diagnostics. The 780 nm system enables rapid intraoperative collagen visualization, effectively reducing diagnostic uncertainties in fibrosis when combined with conventional histology. In parallel, the 820 nm imaging modality achieves sub-micron resolution in quantifying collagen ultrastructural features (e.g., fibril orientation and cross-linking density), thereby refining microenvironmental analysis in chronic fibrotic diseases. Together, these complementary methodologies establish a unified diagnostic framework that integrates clinical efficiency with research-grade precision, advancing pathological practice through real-time decision support and matrix biology studies via mechanistic insights into ECM remodeling dynamics.

## 2. Materials and Methods

### 2.1. The EVG (Elastica Van Gieson) Staining

All animal experiments were performed in strict compliance with institutional animal care guidelines and approved by the Biomedical Ethics Committee at Sun Yat-sen University (Guangzhou, China).

The Elastica van Gieson (EVG) staining technique serves as a histochemical method specifically designed to visualize elastic and collagen fibers in tissue sections under microscopy. Concerning the histopathological evaluation of fibrosis in mouse models—unilateral ureteral obstruction (UUO), folic acid-induced fibrosis, and bleomycin-induced lung fibrosis—EVG staining was performed as follows: In the UUO model, male C57BL/6 mice were anesthetized and subjected to left ureter ligation, with tissue harvested at 5 and 7 days post-surgery. For the folic acid model, mice received intraperitoneal injections of folic acid (250 mg kg^−1^ in 0.3 M NaHCO_3_), and kidneys were collected at 14 days. Moreover, in the bleomycin-induced lung fibrosis model, mice were injected intraperitoneally with bleomycin (500 ng mL^−1^), and lung tissues were harvested at 28 days.

The tissues were fixed in 4% neutral buffered formalin, dehydrated through an ethanol gradient, cleared in xylene, and embedded within paraffin. Sections (5–8 µm) were cut through employing a microtome, mounted on slides, and then dewaxed. After rehydration, elastin fibers were oxidized with potassium permanganate (KMnO_4_) and neutralized with oxalic acid. Staining with the reagent (picric acid and fuchsin) of Van Gieson yielded black/dark brown elastin fibers and pink/red collagen fibers, which enabled clear differentiation of extracellular matrix components.

### 2.2. Identify Regions of Interest by Grid-Targeted Method

Multiphoton microscopy (MPM) imaging of a 5 mm^2^ tissue area (6 µm thickness) requires 1–2 h, which underscores the necessity of pre-identifying regions of interest (ROIs). In systems lacking brightfield imaging, specimen localization is indispensable for providing morphological context, guiding ROI navigation, and allowing spatial registration of fluorescent signals. Due to the absence of brightfield references, a precise correlation between fluorescent markers and tissue architecture is complicated, especially in specimens with sparse or heterogeneous fluorophore distribution [36]. Accurate spatial positioning ensures signal specificity validation, optimal z-plane selection, and multi-channel alignment in topographically complex tissues.

**Figure 1:**
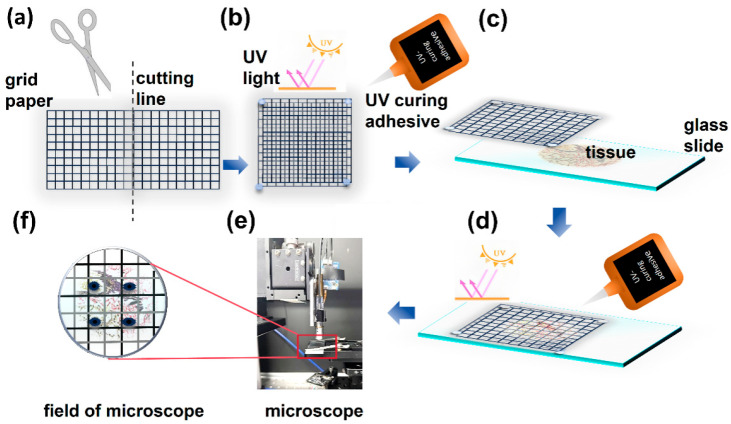
Fabrication of a translucent grid-based marker for precision spatial localization in MPM. (**a**) Fabrication of primary grid units. (**b**) UV-adhesive assembly of the staggered composite grid. (**c**) alignment for composite grid-tissue integration. (**d**) Fixed composite grid on the tissue section. (**e**) Composite-guided ROI localization. (**f**) Magnified view of precision marking on a composite grid.

A grid-targeted method was implemented to identify ROIs precisely. Transparent grid paper was carefully cut into two 1 cm × 1 cm square units (Figure 1a), each comprising a detailed grid of 100 identical 1 mm × 1 mm subgrids. Through leveraging a specialized ultraviolet-curable adhesive, these units were staggered and bonded and created a composite grid with staggered subgrids of 0.5 mm × 0.5 mm (Figure 1b). This configuration effectively reduced the observable field of view under optical microscopy and thus enhanced spatial resolution for ROI localization. To ensure stability during imaging, the assembled grid was securely attached beneath the sample slide (Figure 1c,d). Under optical microscopy, the target position was initially approximated by identifying the corresponding subgrid. The adjacent location was then precisely marked using a fine-tip marker under an optical microscope for further experimental analysis (Figure 1e).

**Figure 2:**
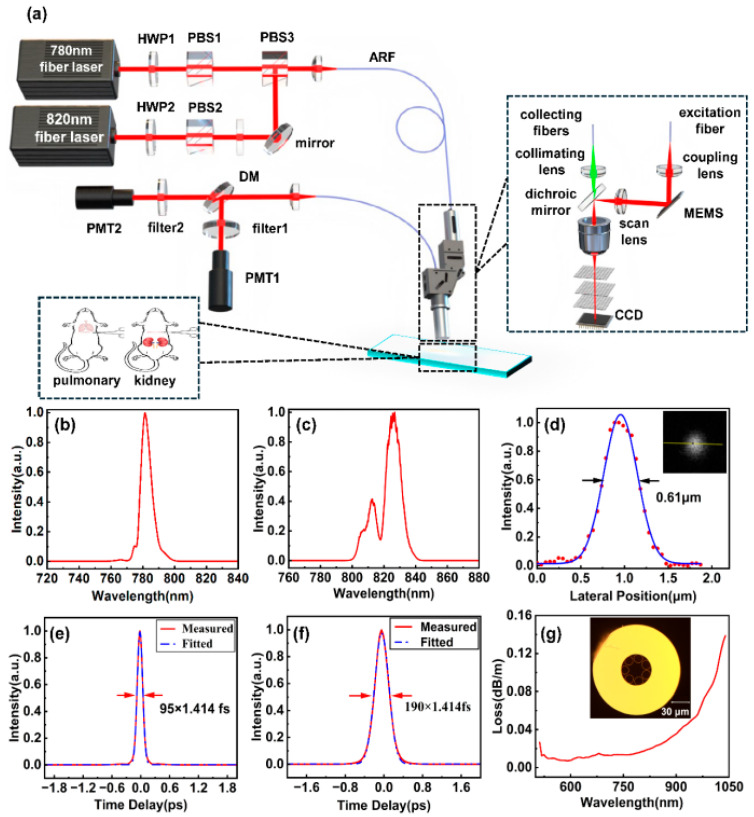
The experimental setup. (**a**) The 780 nm and 820 nm femtosecond lasers, with optical power adjusted by a set of PBS and HWP, are coupled into the ARF through polarization beam combining. The miniaturized probe, scanned by a MEMS, performs two-photon and harmonic imaging on stained sections of the mouse lung and kidney tissue. Auto-fluorescence and second-harmonic signals are separated by a dichroic mirror and individually collected by the PMTs. (**b**,**e**) The 780 nm femtosecond laser spectrum and auto-correlation trace. (**c**,**f**) The 820 nm femtosecond laser spectrum and auto-correlation trace. (**d**) The lateral resolution of the 100 nm fluorescent microb. (**g**) Transmission loss of the antiresonant fiber in the 600–1000 nm range and optical microscopy image of the fiber cross-section.

Following pre-processing, precise alignment between the imaging area and marked ROI was achieved through the coordinated adjustment of the sample stage and imaging probe. Equipped with scan lenses and a Micro-Electromechanical Systems (MEMS) mirror, the probe allowed high-speed planar scanning. Focusing the excitation light onto the sample layer produced a uniform bright red spot on the CCD monitor. The fine positional adjustments of the stage centered this spot within the grid reference markers, ensuring sub-10 μm spatial concordance between the scanning field and fibrotic ROIs. As validated in Figure 2, this calibration protocol established reproducible targeting accuracy and thereby allowed efficient MPM and quantitative analysis of pathological regions.

### 2.3. Preparation of Collagen and Elastin Fiber Samples

Samples of collagen fibers and elastin fibers were prepared for signal detection in both the AF and SHG channels to assess the accuracy and sensitivity of our imaging system. Collagen (C8062, Solarbio, Beijing, China) was first analyzed to remove salt and then freeze-dried by freeze drier (Venus 2.5 L/−90 °C, Furuijie, Shenzhen, China), which was subsequently dissolved in 10 mM phosphate-buffered saline at pH 7.4 to a working concentration of 0.01%. Through leveraging a sodium hydroxide solution, the pH of the solution was adjusted to 7. The prepared solution was then coated onto a glass slide and allowed to dry at room temperature (approximately 20 °C) for several hours (overnight) until a dry and coated surface was obtained. Once fully dry, a lid was placed over the sample, and the gap between the lid and the glass slide was sealed with clear nail polish.

To prepare a 10 mg mL^−1^ solution, the purchased elastin powder (E920946, Mecklin, Shanghai, China) was weighed and dissolved in 10 mM phosphate-buffered saline at pH 7.4. This solution was then subjected to heat treatment at 95 °C for ten minutes. The prepared solution was then coated onto a glass slide and allowed to dry at room temperature (approximately 20 °C) for several hours (overnight) until a dry and coated surface was obtained. Once fully dry, a lid was placed over the sample, and the gap between the lid and the glass slide was sealed with clear nail polish to ensure no leakage or contamination.

## 3. Results

### 3.1. Imaging System Setup and Configuration

The schematic design of the dual-wavelength imaging system is illustrated in Figure 2, comprising the fiber femtosecond light source, hollow antiresonant fiber (ARF), imaging probe, and collection unit. The fiber femtosecond light source outputs two pulses, which are a 780 nm pulse with a 95 fs pulse width and an average power of 50 mW (Figure 2b,e), and an 820 nm pulse with a 190 fs pulse width and an average power of 50 mW (Figure 2c,f). A half-waveplate and a polarization beam splitter (PBS) are used to control and combine the polarization states of the two beams. Additionally, an optical shutter is integrated into the system to enable rapid switching between the beams for efficient sample imaging. The collimated beam is coupled into the probe head via a 1.5 m ARF that has a core diameter of 160 µm and a transmission loss of 50 dB km ^−1^ at both 780 nm and 820 nm. Figure 2g shows the cross-sectional diagram of the ARF. Since this 1.5 m ARF does not introduce significant optical chirp into the femtosecond pulses, no additional dispersive elements were employed to manage optical dispersion. The waveplate and PBS combination, as shown in Figure 2, is utilized to regulate the injection power of the 780–820 nm femtosecond pulses.

The probe head encompasses a Micro-Electromechanical Systems (MEMS) scanner(Mirrorcle, Richmond, CA, USA), a scan lens, a dichroic mirror, an objective, and a collecting lens, as shown in Figure 1a. The MEMS scanner operates at 2400 Hz and yields a frame rate of 9 fps with a frame size of 150 µm × 150 µm. The excitation light is focused by a 9× microscope objective (NA = 0.9) with an average power of approximately 10 mW. When utilizing 820 nm as the excitation wavelength, the resolution, as measured with 100 nm fluorescent beads, is 610 nm (Figure 1d). The generated harmonic signals are collected by the objective, filtered by a dichroic mirror, and delivered to photomultiplier tubes (PMTs) via a silica fiber. To accommodate different detection requirements, two interchangeable filter sets are positioned in front of the photomultiplier tube (PMT). These filters can be rapidly switched to enable flexible signal acquisition across multiple channels. To rapidly locate and detect collagen and elastic fibers, a bandpass filter (F390/40, Jiushi Optics, Shanghai, China) was installed in the SHG channel, while a longpass filter (LP420, Jiushi Optics) was placed in the AF channel. For precise detection of collagen fibers, a customized ultra-narrow bandpass filter (F410/10, Jiushi Optics, Shanghai, China) was installed in the SHG channel, and a longpass filter (LP425, Jiushi Optics, Shanghai, China) was placed in the AF channel.

It is considered that the pre-experiments play a necessary role in ensuring that all three components are coaxial. As exhibited in Figure 1, a quick localization method involves the replacement of the original microscope objective and femtosecond laser source with a collimated semiconductor laser source coaxial with the optical axis of the objective. The collimated semiconductor laser projects a bright red spot onto the Charge Coupled Device (CCD) detection surface, which suggests the marked area in the grid (Figure 1), and the sample grid position is recorded at this point. Along with the sample and grid, the sample stage is moved in 5 mm increments, the CCD images are recorded at each step, and these images are subsequently stacked. If the position of the red spot remains unchanged across this series of images, it can be reasonably concluded that the grid paper, sample, CCD, and microscope objective are coaxial. At this stage, the collimated laser source can be replaced with the microscope imaging probe, thus allowing the commencement of sample imaging. Since this study does not involve dynamic tissue changes and does not require high temporal resolution, multi-frame averaging was employed to suppress random noise and enhance image quality. The acquisition time for a single frame at each excitation wavelength is approximately 0.33 s. For stack imaging, we selected a frame rate of 10 frames per second (fps), which we found to provide an adequate signal-to-noise ratio. Accordingly, the total acquisition time per wavelength is approximately 3.3 s, resulting in a complete dual-wavelength acquisition time of less than 7 s.

### 3.2. Label-Free Visualization of Collagen and Elastin Fibers Utilizing Multiphoton Microscopy

**Figure 3:**
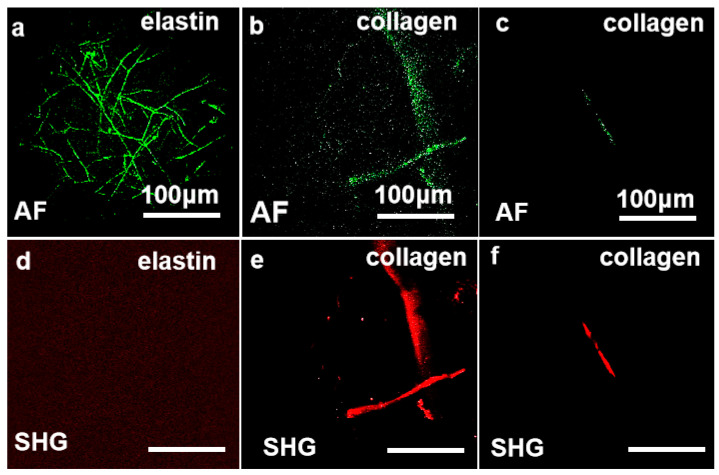
Images of collagen and elastin fibers utilizing the MPM technique system. The method of detection and imaging is indicated in the lower left of each image. (**a**,**d**) MPM imaging of elastin at 780 nm excitation wavelength. (**b**,**e**) MPM imaging of collagen at 780 nm excitation wavelength. (**c**,**f**) MPM imaging methods for collagen at 820 nm excitation wavelength. Green represents the AF signal; red represents the SHG signal.

As illustrated in Figure 3, MPM enables high-fidelity visualization of in situ elastin and collagen fiber architectures. Under 780 nm excitation (Figure 3a,b,d,e), elastin and collagen show AF signatures, while SHG signals are exclusively generated from collagen fibers, which verifies the collagen-specific nature of SHG activity. Conversely, collagen fibers maintain intense SHG emission with attenuated AF signal at 820 nm excitation (Figure 3c,f). These wavelength-dependent contrast mechanisms—AF dominance at 780 nm for elastin and collagen profiling and SHG specificity at 820 nm for collagen mapping—enable label-free and artifact-resistant discrimination and quantitative spatiotemporal mapping of collagen/elastin networks.

### 3.3. Detection of Renal Fibrosis and Pulmonary Fibrosis Utilizing the MPM System

**Figure 4:**
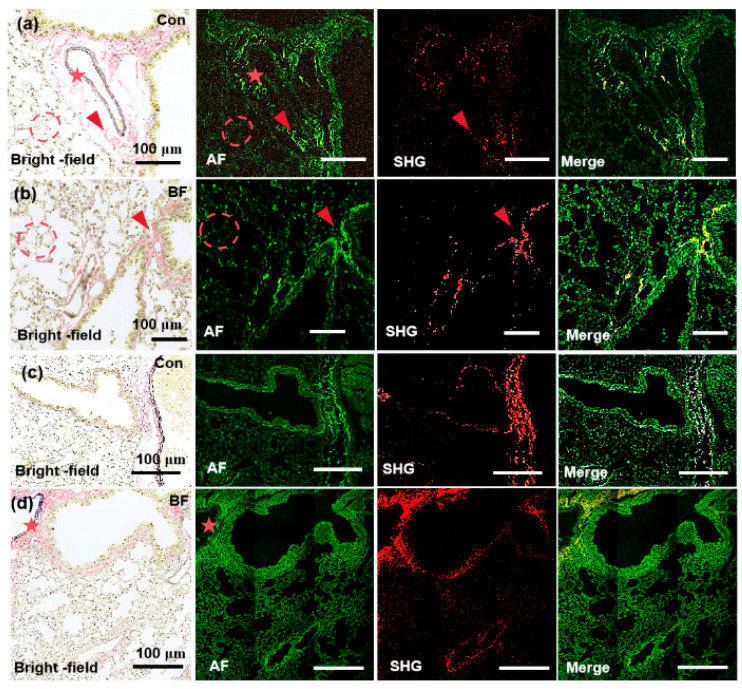
Images of pulmonary tissue specimens selected for the imaging experiments. Imaging modality is noted on the lower left of each panel, with the experimental model labeled on the upper right. (**a**) MPM imaging of pulmonary tissue section in the sham-operated group at 780 nm. (**b**) MPM imaging of pulmonary tissue section in the fibrosis group at 780 nm. (**c**) MPM imaging of pulmonary tissue sections in the sham-operated group at 820 nm. (**d**) MPM imaging of pulmonary tissue section in the fibrosis group at 820 nm. Con—control group, BF—Bleomycin induced pulmonary fibrosis group, the corresponding EVG-stained pulmonary tissues reveal distinct histological features under light microscopy, with collagen fibers stained pink, elastin fibers exhibiting dark purple, and myofibrillar components demonstrating yellow. In the images, the red arrows indicate collagen fibers, red dashed circles highlight alveolar structures, and red stars denote elastin fibers surrounding blood vessels. For multi-channel image analysis, individual channels (e.g., SHG, TPEF) were imported into ImageJ (1.54g, Bethesda, MD, USA) and assigned to specific colors (e.g., red, green) by leveraging the “Merge Channels” function. The final composite images were generated by precisely aligning and integrating the channels, which guaranteed accurate colocalization and signal representation. These high-resolution merged images, saved in TIFF format, enable detailed visualization and quantitative analysis of complex biological structures and facilitate robust interpretation of ECM.

**Figure 5:**
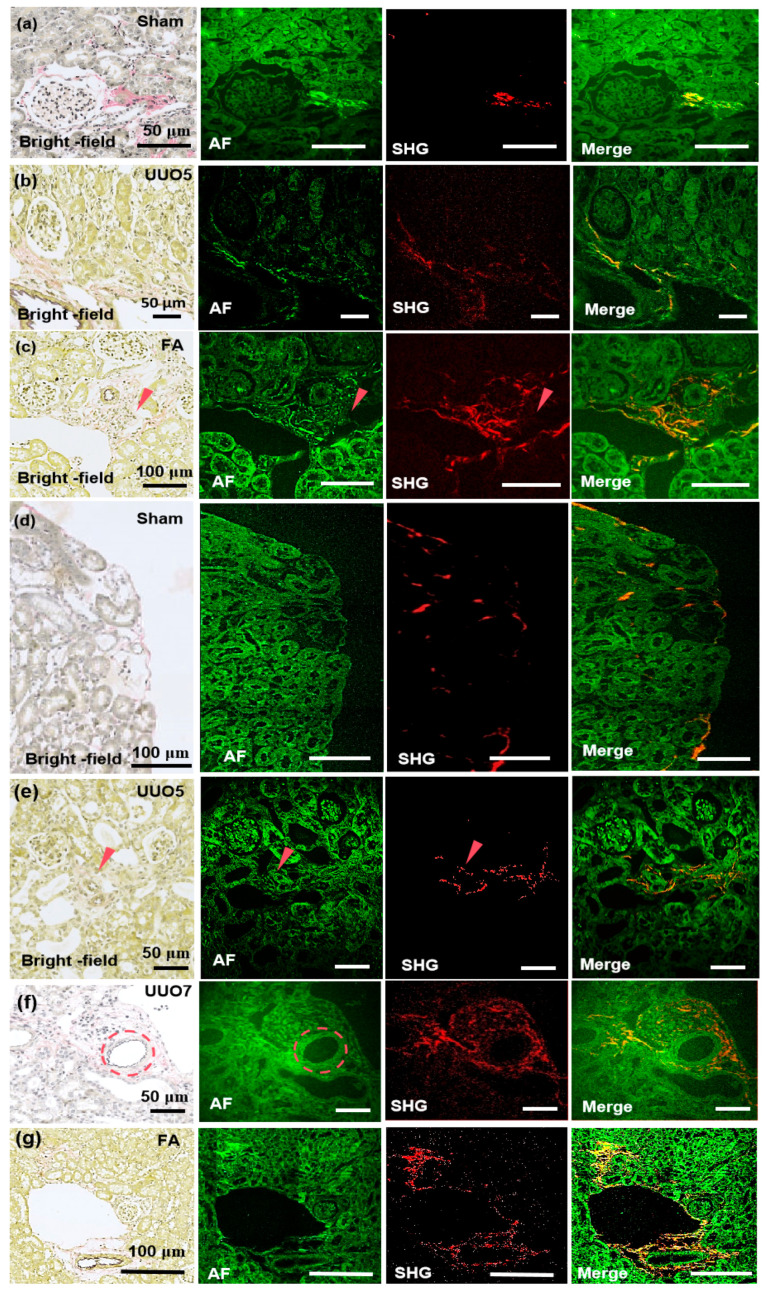
Images of renal tissue specimens selected for the imaging experiments. Imaging modality is noted on the lower left of each panel, with the experimental model labeled on the upper right. (**a**–**c**) MPM imaging of kidney tissue sections in the sham-operated group, renal tissue sections in the 5-day UUO model, and renal tissue sections in the folic acid model at 780 nm. (**d**–**g**) MPM imaging of kidney tissue in the sham-operated group, renal tissue sections in the 5-day UUO model, renal tissue in the 7-day UUO model, and renal tissue in the folic acid model at 820 nm excitation wavelength. Sham—sham-operation group; UUO5—UUO5-day renal fibrosis group. UUO7—UUO7-day renal fibrosis group; FA—folic acid-induced renal fibrosis group. The red arrows represent collagen deposits and areas, and the red dotted circles indicate elastin fibers. For multi-channel analysis, channels were imported into ImageJ, assigned colors, and merged by leveraging “Merge Channels”. The high-resolution composite images, saved as TIFFs, enable detailed visualization and analysis of biological structures, which contributes to ECM interpretation.

In this study, the MPM system was utilized to acquire high-resolution and label-free images of lung and renal fibrosis tissue sections, and the results were systematically compared with conventional histopathological EVG staining to validate both the accuracy and reliability of MPM-based ECM characterization. All imaging experiments were independently replicated three times under identical conditions. Representative images were selected based on consistency with quantitative analyses across biological/technical replicates. The EVG-stained pulmonary tissue sections in Figure 4 and Figure 5 reveal distinct histological profiles under light microscopy, which are collagen fibers (pink), elastin (dark purple), and myofibrillar components (yellow) [37]. Collagen is predominantly localized near vascular structures and constitutes a significant component of alveolar walls, which forms a basement membrane with elastin essential for physiological alveolar expansion and retraction. In the control tissue section (Figure 4a), loosely organized collagen fibers (red arrow) exhibit robust AF signals at 780 nm and significantly exceed elastin-derived AF signals in alveolar walls and perivascular regions. However, the SHG signals at 780 nm only partially overlap with EVG and AF profiles, which implies limited sensitivity for full collagen network characterization. In pathological specimens with pulmonary fibrosis section (Figure 4b), dense fibrotic lesions (red arrow), disrupted alveolar architecture (dotted circle), and thickened septa are demonstrated. While collagen AF signals at 780 nm still play a dominant role in these regions, SHG signals again fail to completely align with EVG/AF markers, which further confirms suboptimal resolution at this wavelength.

At 820 nm (Figure 4c,d), collagen AF signals show a diminishing trend, which presents negligible contrast against alveolar structures, whereas weak AF signals persist in extravascular elastin (red pentagon). Notably, SHG imaging at 820 nm achieves near-complete spatial overlap with EVG/AF-defined collagen fibers, especially in perivascular regions with the dense aggregation of pathological collagen. These results demonstrate wavelength-dependent collagen detection. The 780 nm excitation wavelength enables precise AF-guided localization of collagen microarchitecture. At 820 nm, SHG achieves fiber-level specificity for resolving pathological fibrotic networks. Owing to this dual-wavelength approach, comprehensive visualization of collagen dynamics in pulmonary fibrosis sections is facilitated, balancing sensitivity (780 nm AF) and precision (820 nm SHG) for advanced pathological analysis.

Figure 5a presents the imaging of the renal tissue section from the sham-operation group at 780 nm. The glomeruli exhibit normal size, in which minimal collagen fibers are presented at the glomerular capsule, and the renal tubules appear normal. The AF signal at the collagen site is particularly intense under 780 nm excitation, which correlates well with SHG and EVG staining (pink). In Figure 5b, the imaging of renal fibrosis tissue section from the unilateral ureteral obstruction (UUO) 5-day group under 780 nm excitation is illustrated. The glomeruli are markedly atrophied, the renal tubules demonstrate slight dilation and edema, and interstitial collagen is evident from EVG staining (pink) and SHG. In addition, a small amount of collagen is significantly detectable under 780 nm excitation AF. Figure 5c presents the imaging of the renal fibrosis tissue section induced by folic acid at 780. Folic acid induction reveals atrophied renal tubules, glomeruli nearly filling the entire renal capsule, and increased interstitial collagen fibers beside the renal tubules, accompanied by diffuse collagen deposition. Under 780 nm excitation, the AF signal of collagen protein fibers is visible, which is more distinct than the collagen fibers represented by pink EVG staining, and its SHG signal is also detectable.

The imaging of renal tissue sections from the sham-operation group under 820 nm excitation is shown in Figure 5d. A minimal amount of collagen is present at the cortical edge, the renal tubules are not dilated, and there are hardly any collagen fibers in the renal interstitium. Both SHG and AF can detect these features. Figure 5e presents the imaging of the renal fibrosis tissue section from the UUO 5-day group at 820 nm. According to EVG staining, atrophied glomeruli and slightly dilated renal tubules exist in the UUO 5-day group. The collagen distribution is unidentifiable due to the pink EVG staining at the red arrow is rather indistinct. The AF signal intensity at 820 nm is not particularly prominent and does not detect a strong fluorescence AF signal as observed with 780 nm excitation. However, a clear SHG signal is detectable and shows the distinct arrangement of collagen. Figure 5f displays the imaging of the renal fibrosis tissue section from the UUO 7-day group at 820 nm. Prominent sawtooth-shaped elastic fibers are observed around the blood vessels marked by the dashed circle, with collagen protein fibers accumulating around the vessels and significant dilation of the renal tubules. In the AF channel, corresponding detection of sawtooth-shaped elastic fibers is possible and indicates the potential of this system to detect trace levels of elastic fibers.

Moreover, SHG reaffirms the sensitivity of the 820 nm system in SHG detection as it detects the distribution of collagen fibers at the sub-micron level. Figure 5g shows the imaging of the renal fibrosis tissue section induced by folic acid at 820 nm. The glomeruli are significantly atrophied, the renal tubules are remarkably dilated, and deposited collagen accumulates around the elastic vessels and diffuses into the interstitium. Despite the absence of a detectable differential signal in the AF channel, SHG accurately detects the distribution of collagen fibers. This translation maintains scientific rigor, emphasizes modality-specific findings (AF versus SHG versus EVG), and underscores wavelength-dependent performance differences, which guarantees both logical continuity and technical precision.

## 4. Discussion

Although fibrosis imaging witnesses significant advancements, research on early-stage pathological characterization is still limited. Most studies have focused on tissue-level imaging, primarily because fibrotic markers in bulk tissues generate stronger signals, which facilitates easier detection [38,39,40,41]. However, this approach overlooks the critical need for early diagnosis, where fibrotic sections typically exhibit low marker content, resulting in weak imaging signals that compromise diagnostic accuracy. Furthermore, comprehensive assessment of disease progression is restricted by the limited availability of specific fibrosis markers. In clinical practice, particularly during intraoperative frozen section analysis, the demand for rapid and precise detection is paramount. While AF imaging, SHG, and other multiphoton microscopy techniques theoretically detect collagen deposition in fibrosis [14,16,42], their clinical implementation confronts critical limitations. Signal attenuation or absence caused by low biomarker density, inadequate tissue processing protocols, or subcellular localization failure compromises diagnostic reliability in heterogeneous pathological specimens. All these challenges need to be better addressed to bridge the gap between theoretical capabilities and clinical utility. While the integration of TPEF and SHG imaging techniques can serve as complementary methods for detecting tissue fibrosis, rapid clinical detection necessitates an initial comprehensive tissue imaging followed by precise SHG imaging to underscore fibrosis distribution. However, subsequent rapid detection can be significantly expedited if the approximate location of the target area can be preliminarily identified during the initial overall imaging.

To overcome these challenges, a custom laser imaging system with 780 nm and 820 nm excitation wavelengths was developed, coupled with a novel localization method for precise region-of-interest targeting. To balance the detection wavelength requirements for both collagen and elastin, we selected the shortest possible excitation wavelength while ensuring sufficient excitation intensity. Compared to traditional near-infrared second-harmonic microscopy, this configuration allows the simultaneous achievement of high two-photon and second-harmonic excitation intensities while maximizing resolution, which enables precise localization of even the smallest collagen and elastin fibers. Through the integration of a cost-effective CCD camera, our system achieves accurate marker identification despite the differences between brightfield and fluorescence imaging. The experimental results demonstrate precise localization of collagen regions, with SHG imaging significantly visualizing fibrous structures. This approach not only addresses the limitations of commercial devices that lack brightfield modes but also offers a cost-effective solution for fibrosis detection. The presented design aims to both reduce system size and enhance convenience, which tackles the growing demand for kidney disease monitoring. To achieve submicron resolution, a miniaturized 9× objective lens with a resolution of 0.61 μm at 820 nm is employed, which allows the distinction of even the smallest collagen structures.

Collagen fibers exhibit the strongest AF signals and allow direct localization at 780 nm, while elastin shows weaker fluorescence. SHG at 780 nm detects collagen in early UUO models. At 820 nm, all components display strong AF signals, with collagen playing the dominant role, followed by myofibrils and elastin. SHG signals at 820 nm are more robust and enable sub-micron collagen detection in renal fibrosis. Early fibrosis shows minimal collagen deposition, with rare elastin emerging. In lung fibrosis models, collagen deposition is observed after 14 days, primarily in peripheral vessels and alveolar walls, with cross-linked elastin indicating developmental relevance. According to the experimental results, our equipment not only achieves sub-micron resolution but also exhibits excellent sensitivity. Current diagnostic approaches predominantly employ collagen-targeted fluorescent probes for early fibrotic pathology detection [20,21]. Label-free AF imaging remains underexplored in this context despite its potential to avoid exogenous labeling artifacts and maintain native extracellular matrix integrity. Our system effectively captures collagen AF signals, allowing for rapid preliminary localization of collagen. Additionally, it was also observed that the SHG signal at 820 nm wavelength is significantly stronger than that at 780 nm. Although the intensity of SHG signals is theoretically independent of excitation wavelength, the use of longer excitation wavelengths can reduce light scattering, thereby improving the signal-to-noise ratio and effectively enhancing SHG signal detection. However, under excitation at 780 nm and 820 nm, the SHG signals do not perfectly correspond to the collagen signal observed at 780 nm. This discrepancy is likely due to the polarization sensitivity inherent to SHG, which limits its ability to comprehensively represent the overall tissue structure.

To address this limitation, future studies will incorporate SHG polarization-resolved imaging to better characterize tissue anisotropy and collagen organization. Autofluorescence signals, on the other hand, also originate from a range of endogenous fluorophores such as those found in connective tissue and muscle fibers, complicating the interpretation and spatial localization of specific structures within the image. To mitigate this challenge, we incorporated morphological analysis as a complementary approach to assist in signal differentiation. In prior validation experiments using epithelial tissue, we leveraged the distinct layered architecture of the skin to aid in signal identification. For instance, collagen fibers are predominantly located near the dermis, whereas autofluorescence is largely confined to the superficial layers of the epidermis. These anatomical and morphological differences provide valuable spatial context that facilitates more accurate identification and localization of collagen structures.

To further enhance specificity, future work will include colocalization studies using immunostaining, enabling a more detailed understanding of the spatial distribution and interaction of major tissue components. Additionally, while preliminary observations suggest that elastin detection is feasible, further validation is required, particularly through longitudinal studies across different stages of fibrosis progression.

## 5. Conclusions

In conclusion, a multiphoton microscopy system driven by a fiber-based ultrafast laser was developed, enabling excitation wavelengths at 780 nm and 820 nm for high-sensitivity nonlinear optical imaging. Through simultaneously capturing SHG and two-photon AF signals, this system allows for the precise identification and detection of collagen and elastin fibers. To enhance sample localization and imaging efficiency under non-fluorescence microscopy conditions, we incorporated a grid labeling technique. Using the grid labeling method, the tissue sections from mouse models of renal and pulmonary fibrosis were analyzed. The results revealed that the collagen signal was the most prominently detected in the 780 nm AF channel and enabled rapid and accurate identification of abnormally deposited collagen within the samples. Additionally, the 820 nm imaging system successfully resolved sub-micrometer-scale collagen signals through SHG, which allowed abnormally deposited collagen in early-stage fibrosis to be easily detected. Elastin deposition was observed in pulmonary fibrosis but not within renal fibrosis, suggesting that elastin accumulation is minimal or absent in the early stages of renal fibrosis.

## Data Availability

The data that support the findings of this study are available from the corresponding authors upon reasonable request.

## Abbreviations

The following abbreviations are used in this manuscript:

AF	Autofluorescence
ARF	Antiresonant Fiber
CCD	Charge Coupled Device
ECM	Extracellular Matrix
EVG	Elastica van Gieson
MEMS	Micro-Electromechanical Systems
MPM	Multiphoton microscopy
NA	Numerical Aperture
PBS	Polarization Beam Splitter
PMT	Photomultiplier Tube
ROI	Regions Of Interest
SHG	Second Harmonic Generation
THG	Third-Harmonic Generation
TIFF	Tagged Image File Format
TPEF	Two-Photon Excitation Fluorescence
UUO	Unilateral Ureteral Obstruction
UV	Ultraviolet
